# A fast algorithm for BayesB type of prediction of genome-wide estimates of genetic value

**DOI:** 10.1186/1297-9686-41-2

**Published:** 2009-01-05

**Authors:** Theo HE Meuwissen, Trygve R Solberg, Ross Shepherd, John A Woolliams

**Affiliations:** 1 Institute Animal and Aquacultural Sciences, Norwegian University of Life Sciences, Box 5003, N1432 As, Norway; 2 The Roslin Institute, Royal (Dick) School of Veterinary Studies, University of Edinburgh, Roslin, Midlothian EH25 9PS, UK; 3 Faculty of Business and Informatics, Central Queensland University, Rockhampton 4702, Queensland, Australia

## Abstract

Genomic selection uses genome-wide dense SNP marker genotyping for the prediction of genetic values, and consists of two steps: (1) estimation of SNP effects, and (2) prediction of genetic value based on SNP genotypes and estimates of their effects. For the former step, BayesB type of estimators have been proposed, which assume *a priori *that many markers have no effects, and some have an effect coming from a gamma or exponential distribution, *i.e*. a fat-tailed distribution. Whilst such estimators have been developed using Monte Carlo Markov chain (MCMC), here we derive a much faster non-MCMC based estimator by analytically performing the required integrations. The accuracy of the genome-wide breeding value estimates was 0.011 (s.e. 0.005) lower than that of the MCMC based BayesB predictor, which may be because the integrations were performed one-by-one instead of for all SNPs simultaneously. The bias of the new method was opposite to that of the MCMC based BayesB, in that the new method underestimates the breeding values of the best selection candidates, whereas MCMC-BayesB overestimated their breeding values. The new method was computationally several orders of magnitude faster than MCMC based BayesB, which will mainly be advantageous in computer simulations of entire breeding schemes, in cross-validation testing, and practical schemes with frequent re-estimation of breeding values.

## Introduction

The recent detection of thousands to millions of SNP markers and the dramatic improvements in high-throughput, cost effective genotyping technology have made it possible to apply marker assisted selection at a genome wide scale, which is termed genomic selection [1]. These authors suggested three methods for the estimation of genetic value from dense SNP marker data, namely GS-BLUP, BayesA, and BayesB. GS-BLUP applies the BLUP approach to the estimation of the effects of the marker alleles, which assumes a normal prior distribution for the marker effects, where the variance of the prior distribution was assumed equal for all the markers. Since an equal variance for each of the marker effects seems unrealistic, the BayesA method extended the GS-BLUP method by estimating the variance of every marker separately, and an inverse chi-square prior was used for the estimation of these variances. In the BayesB method it was assumed that many of the markers will actually have no effect, and the prior distribution of the variances was a mixture of a distribution with zero variance and an inverse chi-squared distribution. In a simulation study where the genetic model included a finite number of loci with exponentially distributed effects, BayesB provided more accurate prediction of genetic value than BayesA, which in turn was more accurate than GS-BLUP.

Although BayesB has the potential for the development of more faithful genetic models, and so seems the method of choice for estimating genome wide breeding values (GW-EBV), its calculation requires the use of computer intensive MCMC techniques. For practical applications and for computer simulations of genomic selection breeding schemes, where many selection rounds and replications are required, it would be advantageous if a much faster algorithm for the calculation of BayesB GW-EBV would be available. Thus, our aim here is to present a fast non-MCMC based algorithm for the calculation of BayesB type estimates of GW-EBV. By using a mixture of a distribution with zero effects and an exponential distribution as a prior for the marker effects, the integration involved in calculating the expectation of the breeding values given the data can be solved analytically, which makes non-MCMC estimation of GW-EBV possible.

## Methods

### The model

We will first develop a model for the estimation of the effect of one SNP. The model will be extended to m SNPs in the fourth section of Methods, where m will be assumed to be much larger than the number of records n. When estimating one SNP effect, we generally do not need to use prior information, since the prior will usually be overwhelmed by the information from the data. However, since we will be extending the model to the estimation of many SNP effects, we will use prior information here. Also, in order to facilitate expansion to multi-SNP models we assume that the SNP considered is a randomly chosen SNP with a random effect, instead of a particular SNP with some particular effect. We will assume that a SNP marker has two alleles, 0 and 1, with allele 1 the reference allele having frequency p. For the purpose of estimating gene effects, the SNP genotypes were standardised to form an n × 1 vector of covariates, **b**, defined for animal i as: (1) b_i _= -2p/SD, if i has homozygous genotype '0_0'; (2) b_i _= (1–2p)/SD if i has heterozygote genotype '0_1'; (3) b_i _= 2(1-p)/SD if i has homozygous genotype '1_1'; where SD = √[2p(1-p)]. Thus, the b_i _are standardised such that their mean is 0 and their variance is 1 in a random mating population. If g is the effect of the SNP in the population, and we assume that it is drawn from a distribution of marker effects with mean 0, then the variance due to the marker is Var(b_i_g) = Var(b_i_)Var(g) = Var(g). Therefore by modelling the variance of the effect of the SNP, we also model the variance directly due to the SNP, irrespective of its frequency. This way of modelling the variance due to the SNP will be discussed in detail in Discussion. It may be noted however that the standardisation of the variance of the b_i _is not essential to our derivations below, *i.e*. when considered more appropriate we could choose the b_i _as -2p, (1–2p), and 2(1-p), respectively. The model for the records denoted by the n × 1 vector **y **is:

(1) **y **= **b***g *+ **e**

where g is the effect of the SNP; and **e **is an n × 1 vector of residuals which are assumed normally distributed with variance *σ*_e_^2^. We will ignore the estimation of an overall mean for now since, even if it were included in the model, its estimate would not affect the equation for the estimation of g since **1**^'^**b **= 0, following from the standardisation of **b**.

### Prior distribution of g

The prior distribution of the SNP effect g, *π*(*g*), is assumed to be a mixture of a distribution with a discrete probability mass of zero and an exponential distribution reflected about g = 0 (see [2]):

(2) π(g)={12γλexp(−λ|g|)for g≠0(1−γ)for g=0

where *γ *is the fraction of the SNPs that are in linkage disequilibrium (LD) with a quantitative trait locus (QTL) and may consequently have a non-zero effect; and *λ *is the parameter of the exponential distribution. As described in the Appendix 1 this may be re-expressed as π(g)=12γλexp(−λ|g|)+(1−γ)δ(g) where *δ*(*g*) is the Dirac delta function. Therefore since the variance of the zero-reflected exponential distribution is 2*λ*^-2^, for m markers the total genetic variance is $\sigma_{a}^{2}$ = 2*mγλ*^-2^. Covariances between marker effects are expected to be zero, because any non-zero covariance will depend on the coding of the marker alleles which is arbitrary, *i.e*. a positive covariance can be changed in a negative one by recoding the marker alleles. The hyper-parameters of the prior distribution (*γ *and *λ*) are assumed known here. If one is willing to assume that a fraction *γ *of the markers are in LD with QTL, the variance per marker in LD with a QTL is *σ*_a_^2^/(m*γ*), and an estimate of the *λ *hyper-parameter is √(2 m*γ*/*σ*_a_^2^).

### The posterior distribution and the expectation of g | y

For the likelihood of the data **y **given the genetic effect g a multi-variate normal distribution is assumed with mean **b**g and variance **I***σ*_e_^2 ^from (2), where **I **is the n × n identity matrix. Thus, the likelihood function is *φ*(**y**; **b***g*, **I**$\sigma_{e}^{2}$), where *φ*(**y**; *μ*, **V**) is the multi-variate normal density function with mean ***μ ***and (co)variance matrix **V**. In what follows the dimensionality of the multi-variate *φ*(.;.,.) will be left implicit from the parameters and will include the univariate density. The summary statistic for g is Y = (**b**^'^**b**)^-1^**b**^'^**y **= **b**^'^**y/**n and, consequently, all information on g contained in **y **is also contained in Y. The variance of Y is *σ*^2 ^= (**b**^'^**b**)^-1^*σ*_e_^2 ^= *σ*_e_^2^/n since **b'b **= n. Therefore, the likelihood function *φ*(**y**; **b***g*, **I**$\sigma_{e}^{2}$) is proportional to the univariate density *φ*(*Y*; *g*, *σ*^2^), as shown in Appendix 2, and so we will use the univariate version of the likelihood function for simplicity. The posterior distribution of g|**y **now becomes:

(3) $$p(g|y)=\frac{\phi (Y;g,\sigma^{2})\pi (g)}{\int_{-\infty }^{\infty }\phi (Y;g,\sigma^{2})\pi (g)dg}.$$

The posterior distribution is not affected by the use of either *φ*(**y**; **b***g*, **I**$\sigma_{e}^{2}$) or *φ*(*Y*; *g*, *σ*^2^) since they are proportional to each other, *i.e*. *φ*(**y**; **b***g*, **I**$\sigma_{e}^{2}$)/*φ*(*Y*; *g*, *σ*^2^) = constant, and this constant is a factor of both the numerator and the denominator. The expectation of g given **y **is (*e.g*. [2]) as *E *[*g*|**y**] = ∫ *gp*(*g*|**y**)*dg *and thus:

(4) $$E[g|y]=\frac{\int_{-\infty }^{\infty }g\phi (Y;g,\sigma^{2})\pi (g)dg}{\int_{-\infty }^{\infty }\phi (Y;g,\sigma^{2})\pi (g)dg}.$$

Both the integral in the numerator and that in the denominator are analytically derived in full in Appendix 1. This results in a closed form for E [g|**y**], which is presented in the Appendix 1 in Equation (B3).

### Extension to m SNPs

The extension to the estimation of the effects of m SNPs is obtained by the use of a modification of the Iterative Conditional Mode (ICM) algorithm [3], which we will call the Iterative Conditional Expectation (ICE) algorithm. The ICE algorithm uses the expectation/mean instead of the mode of the posterior, because (1) the mean of g maximises the correlation between *g *and $\hat{g}$[2]; and (2) due to the spike at zero the posterior may be bi-modal (see Results), in which case the mode may be quite far away from the mean. The ICE algorithm iteratively calculates E(g|Y) for each SNP in turn, using the current solutions of all the other SNPs as if they were true effects, which is known to only approximately converge to the correct solution. We will first describe the algorithm and next its approximate nature. The effects of the SNPs are denoted g_i _(i = 1, .., m), and in vector notation by **g**. When estimating the effect of the i^th ^SNP, the current estimates of all other SNPs are used to calculate Y_i _and *σ*^2^. Iterating from a starting solution, *e.g*. $\hat{g}$ = 0, the algorithm performs within each iteration the following steps:

For all SNPs i = 1, .., m,

Step 1: calculate 'adjusted' records, *y*_-*i*_, which are corrected for all the other SNPs so $y_{-i}=y-\sum_{j\neq i}b_{j}{\hat{g}}_{j}$. Estimate the sufficient statistics $Y_{i}={b^{\prime }}_{i}y_{-i}/n$, and *σ*^2 ^= *σ*_e_^2^/n.

Step 2: Equation B3 of Appendix 1 is used to calculate ${\hat{g}}_{i}$ = *E *[*g*_*i*_|*Y*_*i*_], which is used to update the solution for marker i.

The iteration is stopped when the changes in the solutions become small, *i.e*. (g^q−g^q−1)′(g^q−g^q−1)/(g^q'g^q)<10−6, where subscript q denotes the q^th ^iteration. In the Step 1, it is computationally more efficient to calculate Y_i _directly as $Y_{i}=({b^{\prime }}_{i}y-\sum_{j\neq i}\left({b^{\prime }}_{i}b_{j}){\hat{g}}_{j}\right)/n$. The advantage being that the elements of the normal equations matrix ${b^{\prime }}_{i}b_{j}$ may be stored, which speeds up the calculation of Y_i_.

As mentioned before, if the only fixed effect is the overall mean, no correction for fixed effects is needed. If it is required to estimate fixed effects *β *with design matrix **X**, the calculation of **y**_-i _in the Step 1 becomes $y_{-i}=y-X\beta -\sum_{j\neq i}b_{j}{\hat{g}}_{j}$ and each iteration also updates the solutions for the fixed effects *e.g*. by $\beta ={\left(X^{\prime }X\right)}^{-1}X^{\prime }(y-\sum_{j}b_{j}{\hat{g}}_{j})$.

The approximate nature of the ICE algorithm is due to *y*_-*i *_and thus Y_i _not being known, but being estimated. For ease of notation, this was not denoted by a hat elsewhere in this paper, but in this paragraph it is necessary to make the distinction between the true values of **y**_-*i *_= **y **- Σ_*j *≠ *i*_**b**_*j*_*g*_*j *_and *Y*_*i *_and their estimates: ${\hat{y}}_{-i}$ and ${\hat{Y}}_{i}$, which are calculated as indicated above. Estimation errors in ${\hat{y}}_{-i}$ and ${\hat{Y}}_{i}$ occur due to the estimation errors of the other SNP effects ${\hat{g}}_{j}$ (*j *≠ *i*), which is reflected by their Prediction Error Variance, PEV(*g*_*j*_). From a second order Taylor series expansion of E(g_i_|Y_i_) around its mean ${\hat{Y}}_{i}$, it follows that:

(5) $$E(g_{i}|y_{i})=E(g_{i}|{\hat{Y}}_{i})+0.5\frac{\delta^{2}E(g_{i}|{\hat{Y}}_{i})}{\delta Y_{i}^{2}}Var({\hat{Y}}_{i})$$

where *Var*(${\hat{Y}}_{i}$) is due to prediction error variances and covariances of the g_j_. It follows that *E*(*g*_*i*_|*Y*_*i*_) ≈ *E*(*g*_*i*_|${\hat{Y}}_{i}$), as is implicitly assumed in step 2 of the ICE algorithm, if the second derivative of E(g_i_|Y_i_) is small or *Var*(${\hat{Y}}_{i}$) is small. The latter is probably only the case if there are few markers *j *≠ *i*. The first section of Results investigates the non-linearity, *i.e*. second and higher order derivatives, of the E(g_i_|Y_i_) function.

### Comparison of non- and MCMC based BayesB

The BayesB algorithm developed here will be denoted by fBayesB (for 'fast BayesB') and the BayesB algorithm using MCMC (as described in [1]) will be denoted by MBayesB. fBayesB and MBayesB were compared in twenty simulated populations. The simulation of the population and analysis by MBayesB was conducted by Solberg *et al*. [4] and their paper gives a detailed description of the simulation procedures. In brief, the populations were simulated for 1000 generations at an effective size of 100 in order to create mutation drift balance and LD between the markers and the genes. The genome consisted of 10 chromosomes of 100 cM with a total of 8010 equidistant marker loci including markers at each end of the chromosome. The mutation rate per marker locus per meiosis was high (2.5*10^-3^) to ensure that virtually all the loci were segregating. If more than one mutation occurred at a marker locus, the mutation that resulted in the highest Minor Allele Frequency (MAF) was considered as 'visible', whereas the others were considered 'unvisible' and were thus ignored. With these parameters, the visibility procedure turned 99% of markers into biallelic markers, even if several mutations had occurred, with the remaining 1% being monoallelic. There were 1000 equidistant putative QTL positions, which were chosen such that a QTL position was always in the middle between two marker loci. Whether or not a putative QTL position had an effect on the trait depended on the existence of a mutation during the simulated generations, which occurred with a mutation rate of 2.5*10^-5^. The effects of QTL alleles were sampled from a gamma distribution with scale parameter 1.4 and shape parameter 4.2, and were considered with equal probabilities of being positive or negative.

The scale parameter of the gamma distribution was chosen such that the expected genetic variance was 1 (as in [1]). In generation 1001 and 1002 the population size was increased to 1000, and the animals were genotyped for the 8010 markers. In generation 1001, the animals also had phenotypic records, *i.e*. the phenotype of animal i was:

**y**_*i *_= Σ_*j*_(*a*_*ij*(*p*) _+ *a*_*ij*(*m*)_) + *e*_*i*_

where a_ij(p) _is the effect of the paternal (m, maternal) allele that animal i inherited at QTL locus j; e_i_~N(0, $\sigma_{e}^{2}$), where $\sigma_{e}^{2}$ was set equal to the realised genetic variance in the replicate, such that the heritability was 0.5 in each replicate. Marker effects were estimated using the phenotypes and genotypes obtained from generation 1001. The total genetic values for the animals of generation 1002 were predicted (GW-EBV) from their marker genotypes and the estimates of the marker effects. The accuracy of these estimates was calculated as the correlation between the GW-EBV and the true breeding value, which is known in this simulation study. The coefficient of the regression of the true genetic value on the GW-EBV was used as a measure of the bias of the EBV, and a regression coefficient of 1 implies no bias.

## Results

### The non-linear regression curve

**Figure 1:**
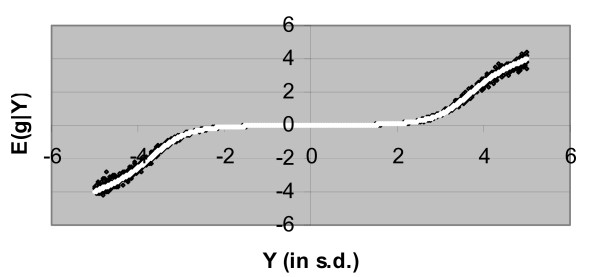
**The expectation of the genetic value given the summary statistic of the data Y, E(g|Y), as a function of Y**. The parameter of the exponential distribution is *λ *= 1, *σ*^2 ^= 1, and the probability of a marker having a true effect is *γ *= 0.05; E(g|Y) calculated by numerical integration is represented by black dots and the analytical solution is shown as white dots.

**Figure 2:**
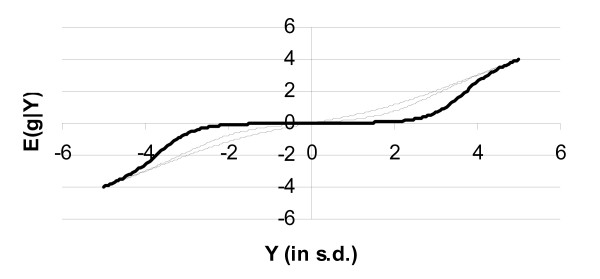
**The expectation of the genetic value given the summary statistic of the data Y, E(g|Y) as a function of Y**. The parameter of the exponential distribution is *λ *= 1, *σ*^2 ^= 1, and the probability of a marker having a true effect is *γ *= 0.05 (**bold curve**), 0.5 (dotted curve), and 1.0 (regular line).

In Figure 1, E [g|Y] is plotted against the value of Y with *σ*^2 ^= 1; since Y is the sufficient statistic for g given the data **y**, E [g|**y**] = E [g|Y]. Figure 1 shows also the regression curve when the integrals in Equation (4) were numerically evaluated, as was done by Goddard [2]. The empirical curve of Goddard has similar characteristics, which is relatively flat at Y = 0, but approaches a derivative of 1 for extreme values of Y. However as a result of the closed expression in Appendix 1 (B3) it is possible to explore the full solution space and Figure 2 shows some examples from this space. The examples demonstrate several features. Firstly E [g|Y] is an odd function (in a mathematical sense) satisfying E [g| -Y] = -E [g|Y]. Secondly, *d E *[*g*|*Y*]/*dY *is non-zero at Y = 0 but decreases towards 0 as *γ *tends to 0. Furthermore *d E *[*g*|*Y*]/*dY *is not necessarily monotonic, for example see *γ *= 0.05 in Figure 2. In the example with *γ *= 0.05 it is clear that *d E *[*g*|*Y*]/*dY *exceeds 1 for Y ≈ 3.5 *i.e*. an increment in Y results in a greater increment in E [g|Y]. Heuristically this occurs because for small *γ *there are only few non zero marker effects, but those present are large; therefore E [g|Y] is close to 0, since Y is expected to have occurred by chance, until Y becomes large and statistically unusual in magnitude, but once considered unusual, E [g|Y] is large. Asymptotically, for Y of large magnitude *d E *[*g*|*Y*]/*dY *tends to 1. The asymptotic behaviour of E(g|Y) is:

$$\text{E}[\text{g}|Y]=\{\begin{array}{ll} Y-\lambda \sigma^{2} & \text{for }Y\to +\infty \\ Y+\lambda \sigma^{2} & \text{for }Y\to -\infty . \end{array}$$

This is nominally independent of *γ*, but for a given *σ*^2 ^the value of *λ *will increase as *γ *increases. Assuming that all the genetic variance can be explained by markers, and the trait has a phenotypic variance of 1 and heritability h^2^, $\lambda \sigma^{2}=(1-h^{2})n^{-1}\sqrt{\left(2m\gamma /h^{2}\right)}$. In summary, the effect is that for small Y values the estimates of g are regressed back substantially, whereas the regression back for large values of Y is diminishing and small.

The non-linearity of E [g|Y] is especially pronounced for small *γ*, and the second derivative seems positive (negative) for positive (negative) values of Y that are approximately between -4 < Y < 4. Since most Y values (expressed in *σ *units) will be between these boundaries, the regression E [g|Y] is quite non-linear. For multiple SNPs, Equation (5) suggests that the estimates of g will be conservative in the sense that they will show a bias towards zero. This bias will be increased for smaller *γ*, *i.e*. for a smaller number of QTL over number of markers ratio.

**Figure 3:**
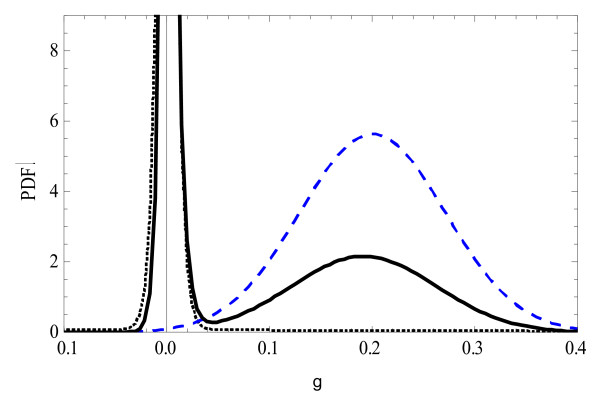
**The probability density function (PDF) of the prior (...), likelihood (_ _ _), and posterior (___) of g**. The parameter from the exponential distribution *λ *= 1.67, *γ *= 0.1, and Y = 0.2 (s.d. units); the spike of the prior distribution at zero is depicted by an exponential distribution, that is reflected around zero, with a very large *λ *of 200.

Figure 3 depicts the prior, likelihood and posterior distribution of g, in a situation where the posterior is bimodal. For smaller values of Y, the likelihood curve moves to the left and the two peaks of the posterior merge into one. For larger values of Y, the likelihood moves to the right, hence its value at 0 reduces, and the peak of the posterior at zero disappears. In the latter case, the posterior becomes approximately a symmetric distribution, and the use of the mode (ICM) instead of the mean (ICE) would not make much difference.

### The accuracy and bias of GW-EBV using BLUP, fBayesB and MBayesB

MBayesB is slightly more accurate than fBayesB (Table 1). The difference in accuracy is 0.011 with s.e. of difference of 0.005, which is statistically significant. For comparison, the accuracy of BLUP GW-EBV is also shown, calculated with a prior that each marker contributes an equal amount of variance, namely 1/m, *i.e*. the infinitesimal model is assumed to hold, at least approximately. The accuracy of the BLUP GW-EBV is considerably lower than that of MBayesB and fBayesB, which is probably because the genetic model underlying the simulations is quite far from the infinitesimal model.

**Table 1:** The accuracy of MBayesB, fBayesB and BLUP, defined by the correlation between true and estimated breeding values in generation 1002.

**Method**	**Accuracy + se**	**Regression + se**
fBayesB	0.849 ± 0.011	1.145 ± 0.025
MBayesB	0.860 ± 0.010	0.923 ± 0.011
BLUP	0.694 + 0.006	0.990 + 0.009

Together with the regression of true on estimated breeding value.

MBayesB and fBayesB incorporate BayesB assumption calculated using MCMC or by the methods described here, and BLUP denotes estimation assuming equal variances due to each of the markers

Both MBayesB and fBayesB yielded biased EBV, but, interestingly, the biases are opposite in direction. MBayesB yields EBV that are too variable, so the EBV require to be shrunk in order to predict the TBV without bias, hence the regression of the TBV on the EBV is <1. In contrast, fBayesB yields EBV with too little variance, so the EBV require to be scaled up in order to predict the TBV without bias, *i.e*. the regression is >1. This conservative behaviour of $\hat{g}$, *i.e*. differences between the estimates are smaller than the real life differences, is expected based on the non-linearity observed in Figure 2 and Equation (5) (see first section of Results). Especially, the bias of the fBayesB is considerable, and should be corrected for by rescaling the EBV when used in a breeding scheme where EBVs based on different amounts of information are to be compared.

**Figure 4:**
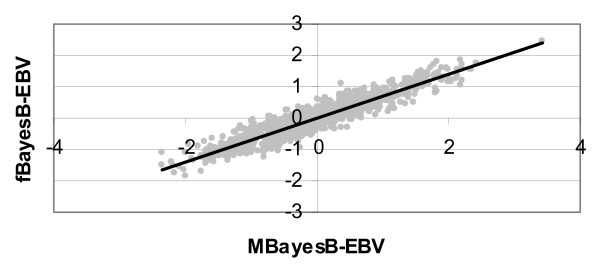
**Scatter plot of fBayesB-EBV against MBayesB-EBV and their linear regression line**.

In order to investigate any systematic deviations of fBayesB-EBV from the MBayesB-EBV, which is considered the golden standard, Figure 4 plots both types of EBV against each other. The regression of the EBV from both types of non-linear regression methods against each other seems pretty linear, which indicates that both methods seem to non-linearly regress the phenotypic data in a very similar way.

### Computer time

The difference in computer time is large: whereas fBayesB takes 2 to 5 minutes MBayesB takes 47 h to compute. No attempt was made to implement parallel computing with MBayesB, although it is readily amenable to parallel procedures through running multiple shorter MCMC chains (each chain should be longer than the burn-in period), which reduces the required wall-time for the calculations. The impact of parallel computing procedures of fBayesB is less clear, but there also appears to be no need for this.

## Discussion

A fast, non-MCMC based algorithm, called Iterative Conditional Expectation (ICE), was derived for the calculation of GW-EBV using dense SNP genotype and phenotypic data. The speed of improvement is due to the analytical integration of the integrals involved in the calculation of E [g|Y]. The Bayesian estimation model used here has very similar characteristics to BayesB as described by [1] and denoted MBayesB here: the prior distribution is a mixture of a heavy-tailed distribution (fBayesB: exponential distribution; MBayesB: a normal distribution whose variance is sampled from an inverse chi-squared) and a distribution with zero effects. The latter mixture distribution is also called a 'spike and slab' mixture [5]. The QTL effects had a Gamma distribution, and thus differed from both that of fBayesB and MBayesB. It may be noted that the prior distribution of fBayesB and MBayesB does not apply to the QTL effects but to the marker effects, for which the true prior distribution will be hard to derive. Although we cannot rule out that the slightly better accuracy of MBayesB compared to fBayesB (Table 1) is due to the prior distribution of MBayesB being better than that of fBayesB, the non-linear regressions of MBayesB and fBayesB seemed very similar since they resulted in a linear regression of the fBayesB-EBV on the MBayesB-EBV (Figure 4). The difference in accuracy seems to be due to that the ICE algorithm ignores higher order derivatives of E(g|Y) function to Y (Eqn. 5). Relaxing this assumption requires (1) taking second order derivatives of the E(g|Y) function, (2) calculation of prediction error variances of $\Sigma b_{j}{\hat{g}}_{j}$, and more research is needed to perform these calculations computationally efficiently.

The justification for the 'spike and slab' prior distribution is that many of the SNPs will not be in LD with a QTL and thus have no effect, whereas the SNPs that are in LD with a QTL have a distribution of effects that is similar to that of the QTL, albeit smaller in magnitude due to the need for several markers to predict the effect of the true QTL genotypes. The true distribution of QTL is often reported to be exponential or gamma [6]. Hayes and Goddard [6] found a shape parameter for the gamma distribution of 0.4, *i.e*. a leptokurtic shape similar to that of the exponential distribution. Where the marker is not in perfect LD with the gene, it will pick up only a fraction of the gene effect and the impact of this on the distribution of marker effects is included within the assumptions concerning their prior distribution.

The non-linear regression curve, resulting from the choice of the prior distribution, is rather flat for values of Y close to 0, but approaches a ratio of E [g|Y]/Y = 1 for Y of large magnitude, so E [g|Y] ≈ Y albeit for very large, and hence rare, deviations. Thus large values of Y are assumed to represent true marker effects, whereas small values are regressed back substantially, *i.e*. are unlikely to represent a true effect. In contrast, if Best Linear Unbiased Prediction (BLUP) of marker effects is used, *i.e*. a normal prior distribution of the marker effects, the regression does not depend on Y and is a constant equal to *σ*^2^/(*σ*^2 ^+ *σ*_m_^2^), *i.e*. E(g|Y) = Y* *σ*^2^/(*σ*^2 ^+ *σ*_m_^2^), where *σ*_m_^2 ^is the variance of the marker effects, which will be *σ*_a_^2^/m. This distinction is due to the use of the normal prior instead of the exponential and, as a consequence, the heavier tails giving credence to large marker effects. Nevertheless the high value of E [g|Y]/Y when using the exponential prior may not be a desirable effect, if outlier data points are encountered.

The variance due to a marker is Var("Marker") = Var(b_i_)Var(g). Here we standardised the variance of the genotypes to Var(b_i_) = 1, *i.e*. the prior variance assumed for the marker effects, g, applies directly for the variance due to the marker, and thus does not depend on marker frequency. We prefer this parameterisation, assuming that the variance of the marker effects is frequency dependent, because (1) QTL with large effects are expected to be at rare frequencies, which implies that the variance of the QTL is roughly constant (at least considerably more constant than when QTL effects were not frequency dependent); (2) if we assume that the QTL variance needs to be above a certain threshold before the markers pick up its effect, these QTL will have a much more constant variance than randomly picked QTL. The algorithm is equally capable of handling the coding of b_i _as 0, 1, and 2, (or after subtracting the mean -2p, (1–2p), and 2(1-p)) for the genotypes mm, Mm, and MM, respectively. The accuracies of the GW-EBV were virtually the same as those in Table 1 when the latter parameterisation of the b_i _was used (result not shown).

The computational advantage of our fast algorithm for the BayesB approach to GW-EBV will not outweigh the reduced accuracy observed, if confirmed for typical trait architecture, when used in practical breeding schemes and the computational time and effort can be afforded. If, in practice, breeding schemes wish to select upon GW-EBV that require frequent updating, then a more appropriate comparison is between frequently updated fBayesB estimates of marker effects and the use of 'old' MCMC based estimates of marker effects, where the GW-EBV of animals are calculated without updating the estimates of marker effects, because of computational constraints. In the latter case there will be a loss of accuracy. It will depend on the amount of new information coming into the breeding value evaluation system, which of these alternatives should be favoured. In simulations of breeding schemes and in cross-validation testing of GW-EBV, the large number of EBV evaluations required may make our fast algorithm the only means to implement BayesB type genome-wide breeding value estimation.

## Competing interests

The authors declare that they have no competing interests.

## Authors' contributions

THEM analysed the data and drafted the manuscript; TRS simulated the data; RS performed literature search on EM type of algorithms and came up with the ICE algorithm; JAW derived the analytical integrations and helped drafting the manuscript. All authors approved the final version of the paper.

## Appendix 1

### Analytical derivation of E [g|Y]

Here we will analytically derive the integrals in (4) in the main text. The prior *π*(*g*) as described in the text is:

(B1) π(g)=12γλexp(−λ|g|)+(1−γ)δ(g)

where *δ*(*g*) is the Dirac delta function . The Delta function has the property that $\int_{a}^{b}f(y)\delta (y)dy=f(0)$ if a < 0 < b and 0 otherwise, consequently $\int_{a}^{b}f(y)\delta (y-c)dy=f(c)$ if a < c < b and 0 otherwise. The numerator of (4) involves an integration from -∞ to +∞, which for the first term in (B1) is here split into:

(B2) $$\int_{0}^{+\infty }g\gamma \frac{\lambda }{2\sigma \sqrt{2\pi }}exp(-\frac{{\left(Y-g\right)}^{2}}{2\sigma^{2}}-\lambda g)dg+\int_{-\infty }^{0}g\gamma \frac{\gamma }{2\sigma \sqrt{2\pi }}exp(-\frac{{\left(Y-g\right)}^{2}}{2\sigma^{2}}+\lambda g)dg$$

The first term in (B2) involves exp(−12(Y−g)2/σ2−λg) and the exponent can be re-expressed as:

−12(Y−g)2/σ2−λg=−12(g−(Y−λσ2))2/σ2+12λ2σ2−λY

The term exp(12λ2σ2−λY) does not involve g and is a constant in the integration, whilst the remaining part, exp⁡(−12(g−(Y−λσ2))2/σ2), is proportional to a normal density with mean (*Y *– *λσ*^2^) and variance *σ*^2^. Now collecting terms, the first term of (B2) becomes:

12γλexp⁡(12λ2σ2)exp⁡(−λY)∫0+∞g(σ2π)−1exp⁡(−12(g−(Y−λσ2))2/σ2)dg

The integrand may be recognised as being proportional to the mean of a truncated normal distribution, where the truncation point is 0, and truncated normal distributed has mean (*Y *– *λσ*^2^) and variance *σ*^2^. The constant of proportionality is the further normalising required for the true truncated density, which is [1 – Φ(0; (*Y *– *λσ*^2^), *σ*^2^)], where Φ(*x*; *μ*, *σ*^2^) is the cumulative normal distribution function with mean *μ *and variance *σ*^2^. Calculating the mean of a truncated normal distribution belongs to the standard tools used in animal breeding (involving the standardisation of the distribution and calculating the intensity of selection; *e.g*. [7]). For brevity, we define here the function Θ_*U*_(*x*; *μ*, *σ*^2^), which represents the mean of an upper truncated normal distribution with mean *μ *and variance *σ*^2 ^truncated at x. After accounting for the scaling the first term of (B2) is seen to be:

12γλexp⁡(12λ2σ2)exp⁡(−λY)[1−Φ(0;(Y−λσ2),σ2)]ΘU(0;(Y−λσ2),σ2)

Using a very similar derivation, the second term of (B2) becomes:

12γλexp⁡(12λ2σ2)exp⁡(λY)Φ(0;(Y+λσ2),σ2)ΘL(0;(Y+λσ2),σ2)

where Θ_*L*_(*x*; *μ*, *σ*^2^) represents the mean of a lower truncated normal distribution with mean *μ *and variance *σ*^2 ^truncated at x. From (B1) the final term of the numerator of equation (4) is ∫−∞+∞g(1−γ)(σ2π)−1exp⁡(−12(Y−g)2/σ2)δ(g)dg which enumerates to 0 following the rules of the Dirac delta-function.

The integral in the denominator of equation (4), follows similar arguments to the numerator but there is no g in the integrand so that the integrals now calculate the probability masses associated with the truncated distributions of their means. The term with the Dirac delta function integrates to:

∫−∞+∞(1−γ)(σ2π)−1exp⁡(−12(Y−g)2/σ2)δ(g)dg=(1−γ)(σ2π)−1exp⁡(−12Y2/σ2)

After carrying out these calculations and some simplification, and denoting *Y*^- ^= *Y *– *λσ*^2 ^and *Y*^+ ^= *Y *+ *λσ*^2 ^the equation (4) in the main text becomes:

(B3) exp(−λY)(1−Φ(0;Y−,σ2))ΘU(0;Y−,σ2)+exp(λY)Φ(0;Y+,σ2))ΘL(0;Y+,σ2)exp(−λY)(1−Φ(0;Y−,σ2))+exp(λY)Φ(0;Y+,σ2))+2(1−γ)(γλ)−1exp(−12λ2σ2)ϕ(Y;0,σ2)

## Appendix 2

### The likelihood of multivariate data can be described by a univariate likelihood function involving the sufficient statistics of the data

The multivariate likelihood of the data, **y**, as described by model (1) is:

(A1) $$\phi_{m}(y;gb,I\sigma_{e}^{2})\propto \exp(-0.5\frac{\left(y-gb{)}^{\prime }(y-gb)\right)}{\sigma_{e}^{2}})$$

where ∝ means equal up to a proportionality constant; g = the effect of the SNP, and **b **is a nx1 vector of covariates obtained from the SNP genotyping. We re-express the term (**y **– gb)'(**y **– **gb**)) as:

(A2) $$\begin{array}{r} y^{\prime }y-2gb^{\prime }y+g^{2}b^{\prime }b\\ =\frac{g^{2}-2g{\left(b^{\prime }b\right)}^{-1}b^{\prime }y}{{\left(b^{\prime }b\right)}^{-1}}+y^{\prime }y\\ =\frac{{\left(g-{\left(b^{\prime }b\right)}^{-1}b^{\prime }y\right)}^{2}}{{\left(b^{\prime }b\right)}^{-1}}-\frac{y^{\prime }bb^{\prime }y}{\left(b^{\prime }b\right)}+y^{\prime }y \end{array}$$

If we use the likelihood (A1) for the estimation of the SNP effect g, than the last two terms in Equation (A2) are constant and thus do not affect the maximisation of the likelihood. Hence, for the estimation of g, (A1) could be expressed as a single-variate density:

$$\phi (g;Y,\sigma^{2})\propto \exp(-0.5\frac{{\left(g-Y\right)}^{2}}{\sigma^{2}})$$

where *Y *= (**b'b**)^-1^**b'y **and *σ*^2 ^= (**b'b**)^-1^$\sigma_{e}^{2}$ are the sufficient statistics of the data.
